# Oral Health and Pathologies in Migrants and Vulnerable Population and Their Social Impact: The Good Practices of the Intervention Model of a University Dental Clinic

**DOI:** 10.3390/ijerph20010353

**Published:** 2022-12-26

**Authors:** Rocío Trinidad Velázquez-Cayón, Ana Isabel Contreras-Madrid, Susell Parra-Rojas, David Pérez-Jorge

**Affiliations:** 1 Clinical Practice Unit, Department of Dentistry, Faculty of Health Sciences, University Fernando Pessoa Canarias, 35450 Las Palmas, Spain; 2 Department of Didactics and Educational Research, Faculty of Education, University of La Laguna, 38200 La Laguna, Spain

**Keywords:** oral health, vulnerable population, migrants, best practices, university dental clinics

## Abstract

Numerous studies have shown the high incidence of diseases affecting oral health in vulnerable populations. The Canary Islands is a region particularly affected by the low income of its inhabitants and a high migration rate. Poor oral health habits and limited access to health care have turned these groups into risk groups. The role of the Fernando Pessoa Canarias University (CDUFPC) dental clinic in the health care of these groups has been an example of good professional practice and a fundamental resource in their health care. The present study aims to identify the profile of pathologies as well as the impact on the oral health of vulnerable population groups served by the CDUFPC. This study was developed between September 2019 and July 2022 with a sample of 878 patients, of whom 267 (30.4%) belonged to vulnerable groups referred by institutions and social organizations. The results identified the prevalence of dental caries as the main pathology and the lack of good oral habits and commitment to oral health and care.

## 1. Introduction

The World Dental Federation (FDI) defines oral health as “multifaceted and includes the ability to speak, smile, smell, taste, touch, chew, swallow, and convey a range of emotions through facial expressions with confidence and without pain, discomfort, and disease of the craniofacial complex” [1]. Oral health is part of general health, according to the definition of health in [2], and is not only related to the areas of physical, emotional, psychological, and socioeconomic well-being. In this sense, [3] found oral health to be “the first step to well-being”, not only as a determining factor of the quality of human life but also as a factor associated with the appearance of systemic pathologies that can affect the life of the patient at both the individual and socio-health levels.

In Latin American countries such as Spain, oral diseases are considered a public health problem because of their high prevalence. Among these diseases, malocclusions occupy third place in frequency, behind only dental caries and periodontal disease [4].

Common lesions and conditions, such as fibrous lesions, benign migratory glossitis, oral candidiasis (acute–chronic), papillomas, ulcers, and vascular lesions, are unknown to the general population because their clinical manifestations tend to go unnoticed in comparison with those of dental caries or periodontal disease, which are not usually given importance, despite their implicit risk and consequences on general health.

Regarding the etiology of caries and periodontal disease, both are related to the action of bacterial plaque on the teeth and the supporting tissues that surround them: gingiva, the periodontal ligament that attaches them to the bone, and the bone itself [5,6,7,8]. Bacterial plaque is a biofilm composed of bacteria, saliva, food debris, and dead cells. In the case of dental caries, the action of the acids produced by these bacteria attacks the hard tissues that make up the tooth (enamel and dentin), causing their decalcification and subsequent destruction. If the acid action on these tissues advances to the deep and internal tissues of the tooth (intradental vasculonervous bundle or dental pulp), it will cause inflammation, necrosis, and infections [9].

Because we know the relevant role of bacterial plaque in the etiology of dental caries and periodontal disease, we understand the importance of education and awareness for the acquisition of hygiene and dental care habits. Oral hygiene is fundamental, especially from an early age, because of its importance in the consolidation of habits that translate into practices to achieve and maintain a healthy life [9]. In this sense, the population’s level of knowledge about the risks of dental caries and periodontal disease continues to be low; dental caries is so common that they are not recognized as an infectious disease caused by microorganisms, as they actually are [10]. According to [7], nearly 3.5 billion people worldwide have oral conditions, with caries being one of the most common conditions in permanent teeth and in children (520 million suffer from caries in deciduous teeth). The high morbidity incidence of these diseases represents a problem that generates enormous costs for the social and health care system of the different countries. In addition to affecting the oral health of those who suffer from them, they accompany them throughout their lives, causing pain, discomfort, disfigurement, and even death, as pointed out by [2]. The data are particularly noticeable in the case of vulnerable or different-ethnicity populations [11,12,13].

In Spain, the authors of [14] conducted a study to determine the data on the oral health of the Spanish population, including adolescents and the elderly. The results of this study indicated that 95% of the Spanish population was affected by caries and that this figure rose to 100% in older adults, which explained the total tooth loss in this population group. In the case of children under 6 years, 31% suffered from unhealed dental caries in 80–90% of the cases. In total, 30% of adolescents presented dental caries, while the percentage rose to 90% in young adults.

Considering the multifactorial origin of the two main diseases affecting the oral health of the world’s population, we advise that they are perfectly preventable conditions [15]. The consolidation of preventive oral health behaviors and the acquisition of healthy habits should begin at an early age to prevent caries and periodontal diseases from becoming established and remaining active in the oral cavity, causing premature loss of permanent teeth [9].

In 2019, the authors of [16] proposed the adoption of alternative educational models developed for the early childhood education stage because studies [17,18,19,20,21] have found that they favor the promotion of well-being and the quality of life, personality development, and health and contribute significantly to the improvement of individual health and well-being [11,12,13,14,15,16,17,18,19,20,21,22].

Although, as we have seen so far, the general quality of oral health of children, young people, and adults in Spain presents worrying figures, the situation of vulnerable groups of low socioeconomic status (ethnic minorities, migrants, or the rural population) has not aroused as much interest, as evidenced by the scarce research on the subject [22]. Among these vulnerable groups [23,24], homeless and/or poor people and migrants can be found [25,26,27,28].

The authors of [29] proposed a theoretical model based on the model of [27] to explain the origin of this vulnerability, and they highlight three fundamental aspects: lack of resources (low income, difficulty in accessing education, marginalization, scarce social support), unhealthy lifestyles, and high morbidity and mortality rates in relation to their health status.

When we speak of vulnerability in relation to health, we refer to the vulnerable population or group that is more prone than others to develop health problems, either because it is exposed to risk or because of its poor physical, psychological, or social condition, which makes it more sensitive to disease [27]. These groups show special living conditions, poor oral hygiene habits, and a lack of knowledge of the short- and long-term health consequences. These characteristics predispose them to suffer from various conditions such as fibrous lesions, benign migratory glossitis, oral candidiasis (acute–chronic), papillomas, ulcers, vascular lesions, and others.

In its 2012 proposal for intervention policies, a scientific commission for the study of social inequalities in health in Spain indicated that social inequalities in health, low social and economic status, and scarcity of environmental resources were the origins of the vulnerability and manifestation of multiple diseases [30,31], including oral diseases.

The authors of [32] evaluated the link between socioeconomic status and oral health-related quality of life as a function of age. The findings indicated that the presence of oral problems in people at low socioeconomic levels is significantly high in relation to those at medium or high levels.

A good example of this is the case of the Cartuja and Almanjáyar neighborhoods, two neighborhoods in Granada (Spain) whose oral health inequalities are attributed to socioeconomic differences, unhealthy habits, and poor diet [33]. The intervention proposal for the promotion of oral health conducted by the public health system, in coordination with an early childhood and primary education center in the area, resulted in a significant improvement in knowledge and modification of eating habits, as well as oral hygiene. After 18 months of program implementation, a significant increase in oral health knowledge and healthy food consumption was observed, which was clearly manifested in a decrease in the consumption of pastries and sugary soft drinks. Evidence of oral health interventions in disadvantaged socioeconomic contexts has demonstrated a positive effect on the improvement of knowledge and healthy habits.

Currently, a higher incidence of diseases affecting oral health has been reported in disadvantaged populations, with a high prevalence of pain/discomfort, loss of masticatory function, and aesthetic and phonatory problems [32,33,34]. Ref. [35] presented a longitudinal study with people over 50 years of age after analyzing a sample of 14,000 participants over two years and noted the relationship between dental care and economic hardship in households. Families with economic difficulties sacrificed the oral health of all their members. They thus confirmed the modulating effect of economic status on oral health care. Most oral conditions have a multifactorial etiology (biological, social, economic, cultural, and environmental factors) [9,22]. Poor oral health habits and limited access to health care have been described as the main factors associated with the low quality of oral health and with the prevalence of dental caries and periodontal disease in this group [36,37,38,39,40].

When included in oral health care, oral education is the fundamental pillar of prevention since it promotes changes in habits and behaviors that have a direct impact on the oral health of individuals, with important implications for the economic cost to society [2]. In this sense, the authors of [37] considered the need to intervene in three fundamental pillars: (a) frequency in the use of dental services, (b) education in proper oral hygiene, and (c) reduction of the consumption of sugary foods. Their vulnerable situation and the difficulties of migrants, refugees, or socially disadvantaged groups in accessing healthy, quality, and low-sugar foods is a real handicap. Curious as it may seem, it is still believed that sugar feeds and kills hunger, and these groups have easy access to low-cost, highly sweetened, processed foods because they are much cheaper than better-quality foods [37].

The 2019 FDI General Assembly concluded with a policy statement on access to oral health care for vulnerable and underserved populations, which presented a commitment to lifelong access to adequate oral health care for the underserved and vulnerable population. This document recommended that dental schools provide special training to students on how to address the complex oral health conditions of these groups. This gave universities as training centers the opportunity to assume a relevant role in community education and to work with interdisciplinary teams in disadvantaged areas or areas at risk of social exclusion.

The University Dental Clinic of the University Fernando Pessoa Canarias (CDUFPC) is an educational institution that is committed to the Canary Islands by way of its dental care services through the clinical practices of students in the last two years of their dentistry degree studies.

The Fernando Pessoa Canarias University (UFPC), when it began to provide clinical practices to its students in 2018, has been an example of good professional practices and the institution’s commitment to the vulnerable population. These results proved the need to know the impact that the good practices of the CDUFPC have had in relation to the detection of diseases such as fibrous lesions, benign migratory glossitis, oral candidiasis, papillomas, ulcers, vascular lesions, etc., as well as their prevention in vulnerable populations.

## 2. Objective

The present study aims to identify the profile of pathologies as well as the impact on the oral health of vulnerable population groups who have difficulty accessing health care and are treated at the CDUFPC.

## 3. Materials and Methods

### 3.1. Sample

A purposive sampling was performed, and the participation was voluntary. Patient characteristics are low-income people or immigrants. In the latter case, patients come to the clinic through specific collaboration agreements with their corresponding watching over associations and/or institutions.

During the period in which this study was developed (from September 2019 to July 2022), the CDUFPC attended to a total of 878 patients, of which 267 (30.4%) belonged to vulnerable groups that accessed the clinic’s services, referred by institutions and social organizations through an agreement with the UFPC. The sample size represented a confidence level of 95% with a margin of error of 5%.

In recent years, this has presented a real challenge and a commitment to the social collaboration of the university with organizations and institutions that welcome and help people from vulnerable groups (see Table 1).

For data collection, participants were asked to sign an informed consent form or, in the case of minors under 18 years of age, obtain the signature of their legal representative in order to authorize access to their medical records and personal and clinical data. The study was conducted according to the guidelines of the Declaration of Helsinki and approved by the Ethics Committee of the European Scientific Institute (ECESI) (protocol HESU12/2021).

### 3.2. Data Analysis

The aim of the descriptive analysis was to identify the profile of the people who attended and received care and treatment at the CDUFPC, as well as the set of pathologies and oral disorders they presented.

A clinical record for each patient was created and kept. In this record, the clinic coordinator recorded the visit, diagnosis and treatment administered, as well as the surgeries or interventions performed in each case. The records were put into a spreadsheet and, then were exported and analyzed by using the Statistical Package for the Social Sciences version 25 (SPSS, v.25, Chicago, IL, USA).

The incidence of the treatments and their impact on the awareness and maintenance of restored oral health were analyzed. In addition, observations were made on the presence of oral mucosal pathologies and general dental health.

## 4. Results

**Figure 1:**
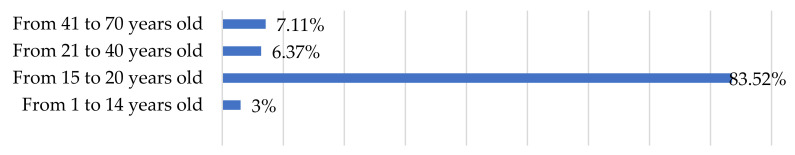
Sample by age group.

We treated 8 (3%) pediatric patients between 1 and 14 years of age, 223 (83.52%) adolescent patients between 15 and 20 years of age, 17 (6.37%) young adults between 21 and 40 years of age, and 19 (7.11%) adults between 41 and 70 years of age. See Figure 1.

The group of pediatric and adolescent patients was made up entirely of migrants, mostly from the African continent (Morocco, Ivory Coast, Gambia, Guinea, Mali, Sierra Leone, Senegal, Mauritania, Cameroon, and Nigeria). The group of young adults was made up of 10 migrants and 7 people referred by the social services of the municipalities belonging to the northern commonwealth of the island of Gran Canaria. The group of adults aged between 41 and 70 years consisted mainly of Canarian patients referred by the social services of the municipalities of Gáldar, Guía, Arucas, and Valleseco, as well as one patient from Senegal. Table 2 shows the origin of the patients treated, regardless of their age.

**Figure 2:**
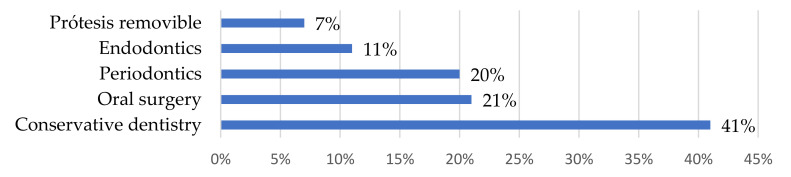
Types of treatments implemented.

Regarding the treatments received, after individual study of each case, 41% involved conservative dentistry (removal of caries of greater and lesser magnitude and obturations), 20% periodontics (professional oral cleaning treatments, oral hygiene education, and basic gum treatment), 21% oral surgery (dental extractions), 11% endodontics (removal of the dental pulp and subsequent filling and sealing of the pulp cavity with an inert material), and 7% removable prostheses (custom-made oral prostheses that can be removed and put in place to replace missing teeth). See Figure 2.

Of note was the detection of 37 lesions related to oral mucosal pathologies that were diagnosed in the adult group (41–70 years of age) and included fibrous lesions, benign migratory glossitis, black hairy tongue, intraoral lesion due to HSV, gingival enlargement due to drugs, florid cemento-osseous dysplasia, and mucosal ulceration with bone sequestration. See Table 3.

It should be noted that in most cases, combined treatments were administered. For example, all patients underwent tartrectomy, excluding only the totally edentulous.

Of the 878 patients seen during this study, 135 treatments were performed with the fabrication of removable prostheses, and only 11 corresponded to people from disadvantaged groups. In the case of removable prosthesis treatments, despite the needs detected, few patients completed their treatment, which demonstrates a low commitment to dental health care. Attendance at the clinic was more a condition for the maintenance of other economic support than a real commitment to oral health care. Only 8.1% of the vulnerable patients who came for treatment with removable prostheses remained in the program.

## 5. Discussion

In order to determine the scope of the CDUFPC in its work and social work, we considered that the academic year 2021–2022 was the first in which the fifth year of the degree in dentistry at the UFPC was completed. Most of the clinical practice hours that the students in training spent with real patients were included in the last year. It should be noted that it has been difficult to follow up on the migrant patients who were referred to the CDUFPC by the centers and institutions; the administrative situation and the lack of interest in the care and maintenance of oral health have been a handicap. Many migrants passed through the dental clinic sporadically or in isolation because, in most cases, they were transferred to centers in other parts of Spain. The pressure from the high occupancy of the migrant reception centers in the Canary Islands makes this a frequent practice; in addition, it should be considered that many of these people were deinstitutionalized or placed back into society early in an attempt to relieve the pressure on the centers and institutions, which means that they were not attended to, evaluated, or treated for the first time for their oral pathologies.

Morocco was the main place of origin for the patients who were treated, and according to recent studies, they are the ones with the worst oral health in comparison with people of other nationalities who migrate to Spain, especially women. Moroccan women reported a greater impact on oral health and quality of life. They also presented greater problems in terms of physical pain and psychological discomfort [39,40,41,42,43].

Among the Moroccan population studied [30,44] in different geographical contexts, it was evidenced that the migrant population reported poor oral health indicators in relation to those of the Spanish population. Oral health problems are a public health concern because of their magnitude, severity, and consequences and because they affect people’s quality of life. These problems are multifactorial and possess biological, social, and contextual characteristics, which were evidenced in the sample of our study [9,29]. In this sense, the findings derived from the Longitudinal Studies of Immigrant Families Project (PELFI) showed how migrants were at a social disadvantage and presented fundamental deficiencies related to dental care and hygiene habits as well as the quality and type of food. Exposure to risk factors has negative effects on oral health that are more evident in these groups than in non-migrants. The incidence of oral pathologies is proportionally higher in the vulnerable population, as evidenced by the data of this study, given that they generally make less use of oral health care because of economic, social, and educational factors (poor training in health care and promotion) or even, in a significant number of cases, because of their lack of commitment and disinterest in oral care. Data such as those provided by [10] indicated an incidence of dental caries of 56.7% in foreign adults between 35 and 44 years of age, compared to 36.7% found in the Spanish population.

It is noteworthy that young people presented alterations whose solution involved conservative treatment, as opposed to older patients whose lesions or pathologies required prosthetic treatment because of missing teeth. In the same sense, the data indicated that the group of migrant patients with conservative dentistry requirements coincided with those reported in the PELFI report, which highlighted the poor oral hygiene education in their countries of origin [37,38].

In the case of older migrants aged 41–70 years who required dental prostheses, the high mobility of the migrants prevented them from undergoing long treatments, such as the fabrication of dental prostheses. This fact was clearly reflected in the data of this study, where only 4.4% of the 250 patients with partial or total edentulous teeth who came for an estimate finally underwent a removable prosthesis treatment. These data seemed to be consistent with those provided by [45,46,47].

Other studies focused on the African population [45,48] have indicated the need to improve oral hygiene education and healthy eating. We should bear in mind that the efforts made by the social and health administrations should be joint if we want to have an impact on greater access to social and health care, including oral health care.

In parallel and coinciding with the results of our study, the studies of [46,47] with the Moroccan population indicated that 90% of children and adolescents had caries. The results associated these data with a low socioeconomic level that generates inequality, evident in both the prevention and treatment of dental pathology.

Regarding the differences in access to oral health care by country, in the meta-analysis presented in 2018, the author of [49] indicated that sub-Saharan Africa was the region with the least access to oral health care. In increasing order, it was preceded by Southeast Asia, South America, North Africa, Asia, Europe, North America, Oceania, and the Scandinavian region. In some countries, oral health care includes the prevention and treatment of existing diseases, while in others, it is limited to prevention only. In many others, there are no oral health programs that include even education or awareness of oral care. The CDUFPC, through its social and health care program for the oral health care of migrants and vulnerable populations, has been developing a fundamental course of prevention and awareness of oral health care. This is essential to improving the quality of life of vulnerable groups, whose chances of receiving care would be practically nil were it not for this type of initiative and program.

Living conditions change radically for people who migrate to another country, especially in the case of people who do not have a support network (family and friends) in the receiving countries. In those countries where there is a support network for these people, prevention and treatment of oral pathologies are considered especially important. As for the sample of our study, the main problems they presented were caries, periodontal disease, oral pain, problems with removable prostheses, gingivitis, and dry mouth [50].

Oral health should be a priority of health care policies, which currently fail to provide continuity and stability of treatment. The lack of economic resources, healthy oral hygiene habits, and vulnerable population’s awareness about the impact of oral health on their quality of life are the main challenges to the care policies that must be developed, especially for the most vulnerable groups.

In 2019, the number of migrants reached 272 million, 51 million more than in 2010 [51,52]. We need institutional involvement for the promotion of policies and actions to improve social and healthcare services for the health and quality of life of this population [9,17,19,29,53].

Along these lines, the CDUFPC has been developing monitoring programs to determine the adherence of vulnerable patients to oral health care and the provision of oral health care for institutionalized elderly people and to promote changes in the oral health care habits in people at risk of social exclusion and raise awareness for the need to monitor and control oral health in vulnerable populations. The actions developed by the CDUFPC are examples of preventive actions necessary for improving the quality of life not only for people in general but especially for the group of vulnerable people. Investment in the implementation of preventive policies on oral health is essential and necessary if we want to offer people acceptable aging and quality of life.

## 6. Conclusions

Initiatives such as the one developed by the CDUFPC during its health care and sanitary work are essential, given the high number of migrants and vulnerable groups on the island of Gran Canaria and the lack of human and economic resources regarding access to oral health care provided by the public health system, which is particularly insufficient and limited in addressing the needs of vulnerable groups.

The CDUFPC carries out important socio-healthcare work by attending to migrants and vulnerable groups on the island of Gran Canaria.

The deinstitutionalization or transfer of centers for migrants prevents the control and follow-up of the treatment given to migrant patients.

The group of people between 15 and 20 years of age from Morocco was found to be the group with the highest incidence of oral pathologies (dental caries and periodontal diseases). They also presented fundamental deficiencies related to dental care and hygiene habits, as well as those related to food quality and type.

In the year 2019, the highest number of incidences attended for oral pathology in the CDUFPC was evidenced. These data coincided with the period prior to the confinement due to the pandemic by COVID-19. This fact, together with the decrease in migratory movements during this period, explained the lower attendance of patients, who were exclusively patients suffering from severe pathologies.

It is essential to implement training and awareness programs on oral care and prevention of oral diseases, especially in the case of migrants and vulnerable populations.

## 7. Study Limitations

Further studies with large samples are needed to confirm the trend of our results in relation to both access to oral health care and awareness of oral health care.

This was the first study conducted in the Canary Islands on the oral health of the vulnerable population. The CDUFPC is the first educational institution to offer comprehensive oral health care treatments for the migrant population in the Canary Islands. It is a relatively young university, having graduated its first class in dentistry in the 2021–2022 academic year, and because it is a new program with a new faculty, weaknesses have been detected in the control and identification of specific aspects of patient anamneses. With the intention of improving and completing this specific information, the research team has developed a clinical protocol that will enhance both the information and the diagnoses of the patients.

Program stability in following up on the efficacy and controlling the evolution of the treatments was one of the main difficulties of this study. We are aware that the conditions and times of access to the program are limited, but we believe that it is possible to implement a follow-up program that allows migrants to continue their treatment and control in the locations to which they are transferred. In this sense, the university is preparing a protocol with health institutions that will allow collaboration in the follow-up of these patients for the maintenance of restored oral health, control of hygiene measures, evaluation of the health of patients who have received treatment, and the continuation and completion of all treatments required by the patient.

## Figures and Tables

**Table 1:** Institutions/organizations having an agreement with the CDUFPC.

Sta. María de Guía Town Hall	Andreas Rosca
Commonwealth of municipalities of the north	White Cross
Mother Africa Canary Islands Foundation	Red Cross
Diagram Foundation	Children’s Villages
Social Quorum	Social Response XXI Century Foundation
Spanish Commission for Refugee Assistance	Life opportunities social association
Canary Foundation of social action m.p. (fucas)	Coliseo Association
Suma Foundation	Doctors of the world

**Table 2:** Patient origin.

Country	N
Morocco	135
Cote d’Ivoire	6
Gambia	22
Guinea	11
Mali	9
Sierra Leone	6
Senegal	13
Mauritania	10
Cameroon	25
Nigeria	2
Venezuela	1
Netherlands	2
Canary Islands	25

**Table 3:** Diagnosed oral mucosal lesions.

	2019	2020	2021	2022	Total
Fibrous lesions	5	2	1		8
Benign migratory glossitis	2		2		4
Oral candidiasis (acute–chronic)	3	2			5
Black hairy tongue	2		1		3
Papillomas	1				1
Prosthetic stomatitis	3				3
Recurrent oral aphthosis (minor/major)	1	1			2
Traumatic ulcers	1				1
Vascular lesions	1				1
Exfoliative cheilitis	1				1
Leukoplakia	2	1			3
Intraoral lesions due to HSV			2		2
Drug-induced gingival enlargement			1		1
Bone cementum dysplasia floridum			1		1
Mucosal ulceration with bone sequestration			1		1
Total lesions	22	6	9	0	37

## Data Availability

Data from the study are available from the lead author of this study upon request.
